# [Corrigendum] MYCN‑amplified neuroblastoma cell‑derived exosomal miR‑17‑5p promotes proliferation and migration of non‑MYCN amplified cells

**DOI:** 10.3892/mmr.2024.13349

**Published:** 2024-10-02

**Authors:** Weiming Chen, Xiwei Hao, Binyi Yang, Yuezhen Zhang, Lingyun Sun, Yanan Hua, Li Yang, Jiabin Yu, Jing Zhao, Lin Hou, Hongting Lu

Mol Med Rep 23: 245, 2021; DOI: 10.3892/mmr.2021.11884

Following the publication of this article, an interested reader drew to the authors' attention that the forward and reverse primer sequences written for GAPDH in Table I on p. 3 were incorrect. Upon requesting an explanation of these errors from the authors, they realized that these sequences had been written incorrectly in the paper: The sequence of the forward primer in Table I should have been written as 5’-CAGGAGGCATTGCTGATGAT-3’, and the reverse primer should have been written as 5’-GAAGGCTGGGGCTCATTT-3’. The Editorial Office also requested seeing proof of purchase of the primers used in this study from the authors.

The authors are grateful to the Editor of *Molecular Medicine Reports* for allowing them the opportunity to publish this corrigendum, and all the authors agree with its publication. The authors also regret the inconvenience that these mistakes have caused.

